# A Moonlighting Enzyme Links *Escherichia coli* Cell Size with Central Metabolism

**DOI:** 10.1371/journal.pgen.1003663

**Published:** 2013-07-25

**Authors:** Norbert S. Hill, Paul J. Buske, Yue Shi, Petra Anne Levin

**Affiliations:** Department of Biology, Washington University, Saint Louis, Missouri, United States of America; Universidad de Sevilla, Spain

## Abstract

Growth rate and nutrient availability are the primary determinants of size in single-celled organisms: rapidly growing *Escherichia coli* cells are more than twice as large as their slow growing counterparts. Here we report the identification of the glucosyltransferase OpgH as a nutrient-dependent regulator of *E. coli* cell size. During growth under nutrient-rich conditions, OpgH localizes to the nascent septal site, where it antagonizes assembly of the tubulin-like cell division protein FtsZ, delaying division and increasing cell size. Biochemical analysis is consistent with OpgH sequestering FtsZ from growing polymers. OpgH is functionally analogous to UgtP, a *Bacillus subtilis* glucosyltransferase that inhibits cell division in a growth rate-dependent fashion. In a striking example of convergent evolution, OpgH and UgtP share no homology, have distinct enzymatic activities, and appear to inhibit FtsZ assembly through different mechanisms. Comparative analysis of *E. coli* and *B. subtilis* reveals conserved aspects of growth rate regulation and cell size control that are likely to be broadly applicable. These include the conservation of uridine diphosphate glucose as a proxy for nutrient status and the use of moonlighting enzymes to couple growth rate-dependent phenomena to central metabolism.

## Author Summary

The observation that growth rate and nutrient availability strongly influence bacterial cell size was made over forty years ago. Yet, the molecular mechanisms responsible for this phenomenon have remained elusive. Using a genetic approach, we identified proteins responsible for increasing *Escherichia coli* cell size under nutrient-rich conditions. Our data indicate that OpgH, a glucosyltransferase involved in cell envelope biogenesis, interacts with FtsZ, a key component of the bacterial cell division machinery. In the presence of a modified sugar, UDP-glucose, OpgH interacts with FtsZ to delay the timing of division machinery assembly. Comparison of the *E. coli* pathway with the parallel *Bacillus subtilis* pathway illuminates a striking example of convergent evolution in which two highly divergent bacteria employ unrelated glucosyltransferases for an essential part of cell cycle regulation and reveals aspects of metabolic and physiological control that are potentially applicable to all forms of life.

## Introduction

Cell size control is a fundamental aspect of the cell cycle. Coordinating cell growth with division is essential to ensure that daughter cells have sufficient room for cytoplasmic and genetic material and are the correct size for a given condition or developmental fate. Despite the universal requirement for size control, how cells are able to detect the achievement of a particular size and communicate this information to the division apparatus remains an unresolved question in cell biology [1].

Nutrient availability is a primary determinant of cell size for single-celled organisms. In their seminal 1958 studies Schaechter, Maaløe, and Kjeldgaard determined that *Salmonella* cell size is coupled to growth rate, which is itself a function of nutrient availability [2]. Later work established that growth rate and nutrient availability are conserved determinants of cell size. *Escherichia coli* and *Bacillus subtilis* both coordinate cell size with nutrient availability, as do single-celled eukaryotes including the classic cell cycle model organism *Schizosaccharomyces pombe* [3], [4], [5].

To coordinate growth rate and nutrient availability with size, cells must have a mechanism to transmit information about growth rate and metabolic status to the division machinery. In *B. subtilis*, cell size is coordinated with central metabolism in part through uridine diphosphate glucose (UDP-glucose)-dependent changes in the oligomerization potential of the glucosyltransferase, UgtP [6], [7]. During growth in nutrient-rich medium UDP-glucose, synthesized in a two-step pathway from glucose-6-phosphate, stimulates interaction between UgtP and the highly conserved tubulin-like cell division protein FtsZ, delaying assembly of the division machinery and increasing cell size. Conversely, during growth in nutrient-poor medium and/or in the absence of UDP-glucose, UgtP favors self-interaction. This permits division to proceed unimpeded yielding a smaller size. UgtP is a moonlighting enzyme also required for synthesis of the diglucosyl-diacylglycerol (Di-glc-DAG) anchor for lipoteichoic acid (LTA), an anionic polymer that is a major component of the Gram-positive cell wall. Loss-of-function mutations in *ugtP* disrupt synthesis of the Di-glc-DAG moiety, but does not impact LTA synthesis [8].

Previous reports have indicated that inactivating UDP-glucose synthesis by inactivating the phosphoglucomutase, *pgm*, results in a ∼25% reduction in *E. coli* cell size under nutrient-rich conditions [9], [10]. This phenotype suggests that *pgm* mutant cells are unable to properly coordinate cell division with nutritional conditions and, furthermore, implicate UDP-glucose as a widely conserved intracellular proxy for nutrient-dependent size control. Interestingly, despite the apparent conservation of UDP-glucose as a signaling molecule, the identity of the effector is less obvious. As a Gram-negative bacterium, *E. coli* does not synthesize LTA and computational analysis does not reveal a *ugtP* homolog within its ∼4.6 MB genome.

Here we report the identification and characterization of the integral inner-membrane protein OpgH as a UDP-glucose-activated inhibitor of FtsZ ring formation in *E. coli*. Genetic and biochemical data indicate that OpgH interacts directly with FtsZ via its N-terminal domain to inhibit division in a UDP-glucose-dependent manner. OpgH is the functional homolog of UgtP, a sugar transferase in *B. subtilis* that modulates cell size in response to carbon availability. While both moonlighting enzymes serve as membrane-associated glucosyltransferases, they share no homology, have distinct enzymatic activities, and inhibit FtsZ assembly through different mechanisms thereby exposing a remarkable instance of convergent evolution. Moreover, this work significantly advances the understanding of nutrient-dependent cell size control in *E. coli*, the predominant model organism for studying bacterial physiology.

## Results

### *E. coli* utilizes UDP-glucose to couple cell size with nutrient availability

**Figure 1:**
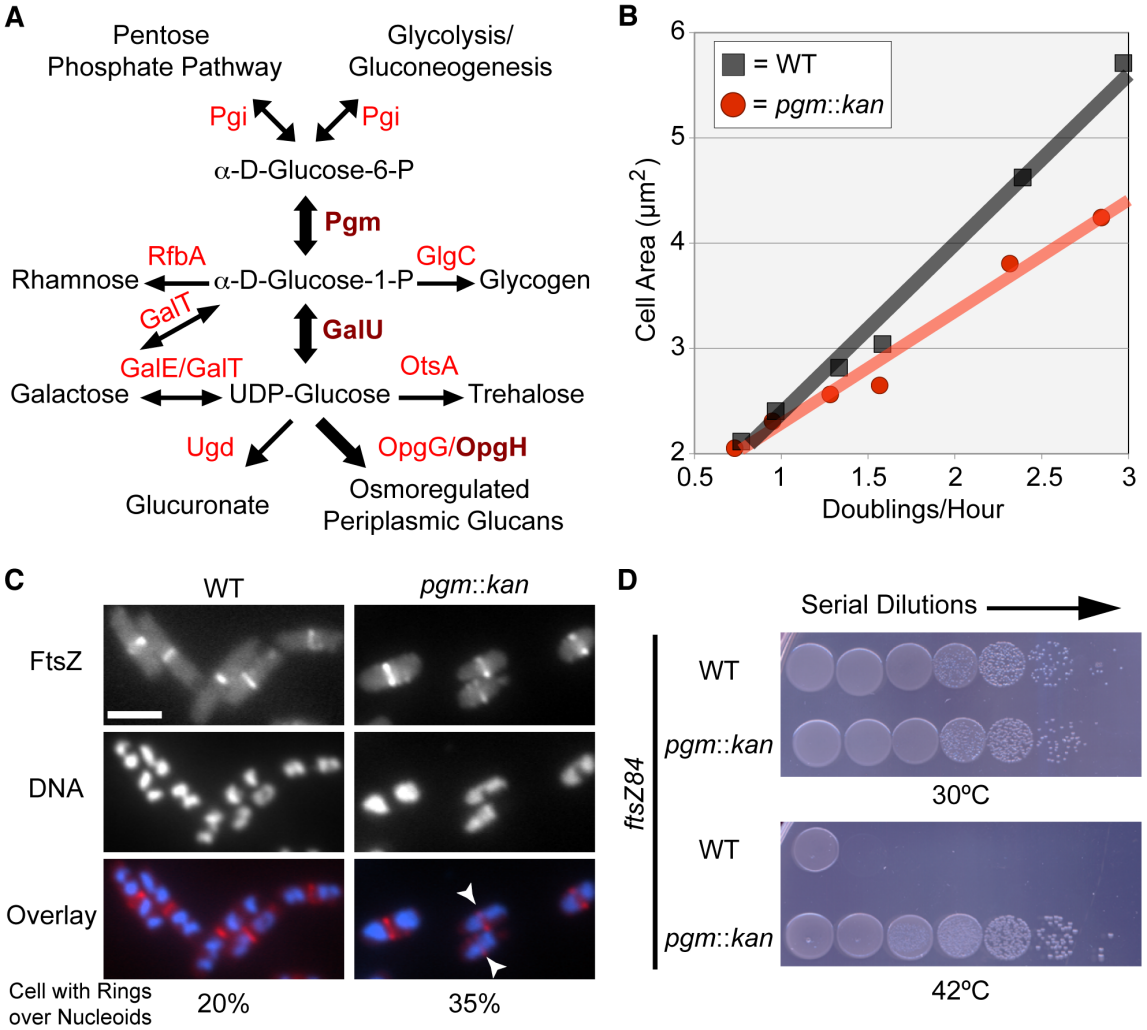
*E. coli* utilizes UDP-glucose to coordinate nutrient availability with cell size. (A) A simplified schematic of UDP-glucose production and utilization in *E. coli*. Substrates are in black font, enzymes are in red font. Enzymes crucial to coordination of size with growth rate are in dark red and bold. Metabolic pathway information was derived from the Kyoto Encyclopedia of Genes and Genomes (KEGG) website [75], [76]. (B) Cell area measurements of wild type (black squares) versus *pgm*::*kan* (red circles) in various growth media (from left to right: AB-succinate, AB-glucose, AB-succinate + casamino acids (CAA), AB-glucose + CAA, LB, and LB-glucose). (C) Occurrence of FtsZ rings over incompletely segregated nucleoids in wild type or smaller *pgm*::*kan* cells. Both strains encode a chromosomal copy of *P_lac_*::*gfp*-*ftsZ* and were cultured in nutrient-rich conditions (LB-glucose with 1 mM IPTG). GFP-FtsZ (top), DAPI stained DNA (middle), and the overlay with FtsZ in red and DNA in blue (bottom). White arrows indicate cells with a division ring over partially segregated chromosomes. Bar = 2.5 µm. (See Table S1 for expanded analysis.) (D) Disrupting UDP-glucose production by disrupting *pgm* suppresses lethality of FtsZ84 in non-permissive conditions (42°C). Serial dilution plating of strains with *ftsZ84* ± *pgm*::*kan* grown on nutrient-rich, low-salt media at 30°C (permissive) or 42°C (restrictive). See also Figure S1 (expression levels of FtsZ84).

Previous work from our lab and others suggests that *E. coli* may employ UDP-glucose as an intracellular proxy for nutrient availability in the regulatory circuit responsible for coupling cell size with growth rate [10], [11]. To test this we examined the size of wild type cells and *pgm* null mutants that are defective in the first step of UDP-glucose biosynthesis (Figure 1A). If UDP-glucose is central to the nutrient-dependent control of *E. coli* cell size, the size differential between wild type and *pgm* mutant cells should be greatest under nutrient-rich conditions and least under nutrient-poor conditions.

Measurements of the cross-sectional area of cells cultured under a range of nutrient conditions indicate that *pgm* mutants are indeed defective in the growth-rate-dependent control of cell size (Figure 1B). In LB+0.2% glucose (LB-glucose), the average cross-sectional area of wild-type *E. coli* was 5.66 µm^2^ while *pgm* mutants were 4.24 µm^2^, a difference of over 25%. However, the size inequity dissipated under nutrient-poor conditions. *pgm* mutants were only 4% smaller than wild type when cultured at a growth rate 4-times slower in a minimal growth medium supplemented with 0.4% succinate (AB-succinate).

### Defects in UDP-glucose biosynthesis increase the frequency of FtsZ rings over incompletely segregated nucleoids

Rapidly growing cells are not only longer, but also have more total DNA, a consequence of multifork replication. Multifork replication is a phenomenon that allows *E. coli* and other bacteria to sustain mass doubling times shorter than the period required to complete chromosome replication and division. In previous work we determined that growth rate-dependent increases in cell size help prevent aberrant assembly of the division machinery over unsegregated bacterial chromosomes (nucleoids) in *B. subtilis* [6]. To determine the impact of a reduction in *E. coli* cell size on the frequency of division rings formed over nucleoids, we visualized FtsZ ring formation in rapidly growing *pgm* mutant cells with an inducible copy of *ftsZ* fused to *gfp* [12].

Consistent with growth rate-dependent increases in cell size ensuring that *E. coli* cells have sufficient room for DNA segregation during multifork replication, we observed a near two-fold increase in the frequency of division rings positioned over nucleoids in *pgm* mutants relative to wild type. In accordance with previous studies [13], approximately 20% (130/654) of wild type cells had an FtsZ ring over chromosomal material. The frequency increased to 35% (246/662) in diminutive *pgm*::*kan* cells (Figure 1C; Table S1). This suggests that the growth rate-dependent size increase is, in part, a mechanism to spatially coordinate the excess DNA generated by multifork replication.

### Defects in UDP-glucose biosynthesis stabilize FtsZ assembly at midcell

To coordinate size with growth rate, cells must evaluate nutrient availability and subsequently transmit that information to the division machinery. To assess the impact of UDP-glucose on FtsZ assembly in *E. coli*, we determined if defects in UDP-glucose biosynthesis were sufficient to suppress the conditional lethality of the heat-sensitive *ftsZ84* allele. FtsZ84 (G105S) supports assembly of the division ring at 30°C, but is unable at 42°C, leading to extensive filamentation and cell death [14], [15].

Consistent with increases in UDP-glucose activating an inhibitor of FtsZ assembly, the viability of *pgm*::*kan ftsZ84* double mutants was ∼4.5-fold higher than *ftsZ84* alone under restrictive conditions (Figure 1D). The plating efficiency (CFU restrictive/CFU permissive) of *ftsZ84* cells grown at 30°C versus 42°C was just 0.013% (±0.001). In contrast, *pgm*::*kan ftsZ84* double mutants exhibited essentially no reduction in viability under identical conditions 99.2% (±0.3). Notably, this is the first example of a loss-of-function mutation that suppresses the heat sensitivity of *ftsZ84* without increasing the intracellular concentration of FtsZ84 (Figure S1). Together, the data presented in Figure 1 provides evidence for a UDP-glucose-activated factor coupling cell division directly to carbon metabolism.

### Defects in the glucosyltransferase OpgH reduce *E. coli* cell size

To identify the UDP-glucose-dependent regulator of *E. coli* cell size, we systematically screened kanamycin resistance cassette insertions in genes predicted to be associated with UDP-glucose synthesis or utilization for defects in cell size, taking advantage of the Keio mutant collection (Figure 1A) [16]. Importantly, mass doubling rates in these mutants were indistinguishable from wild type in both nutrient-rich and nutrient-poor conditions (Table S2).

**Figure 2:**
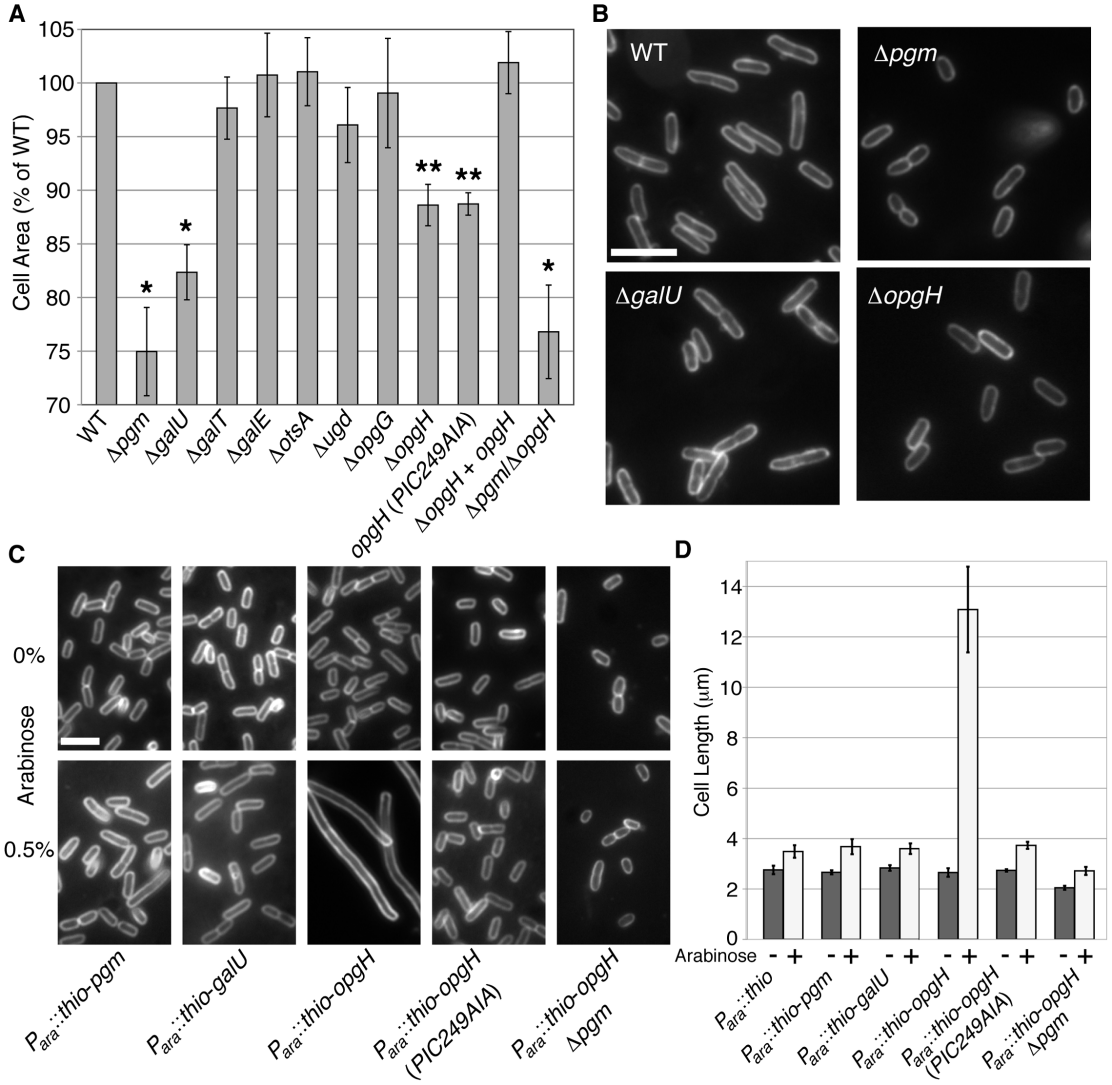
OpgH acts as a nutrient-dependent division antagonist. (A) Cell area measurements of mutants in UDP-glucose synthesis or utilization cultured in LB-glucose. WT is set to 100%. >250 cells were measured per sample, error bars are standard deviation (n>3). The *opgH* null is complemented with a plasmid encoding *P_lac_*::*opgH*-*gfp* and was cultured with 0.08 mM IPTG. This fusion is shown to be functional for glucosyltransferase ability (Figure S5). * denotes p>0.001, ** signifies p>0.05 as judged by chi^2^ analysis. (B) Micrographs of WT and knockouts of genes involved in UDP-glucose pathway grown in LB-glucose and stained with the membrane dye FM4-64. Bar = 5 µm. (C) Representative micrographs and (D) cell length measurements of strains cultured in LB overexpressing genes in the UDP-glucose pathway from an arabinose inducible promoter. Uninduced cells are on the upper panel. Induced constructs are on the bottom panel. Cells are stained with FM4-64. Bar = 5 µm. Error bar denotes standard deviation (n = 3). See also Table S2 (information of length, width, and growth rate), Figure S3 (measurements of cells defective in factors adjacent to the UDP-glucose biosynthesis pathway), and Figure S4 (Thio-OpgH-His overexpression levels).

Of the 16 mutants we screened, knockouts in only three genes *pgm*, *galU* encoding a pyrophosphorylase required for synthesis of UDP-glucose from glucose-1-phosphate, and *opgH* (*mdoH*, b1049, EcoCyc:EG11886, UniProt:P62517) encoding a Family II glucosyltransferase, resulted in a statistically significant reduction in cell size (Figure 2A, 2B; Figure S3). Wild-type MG1655 cells had an average area of 5.66 µm^2^. The *galU*::*kan* and *opgH*::*kan* mutants were 18% (4.66 µm^2^) and 12% (5.01 µm^2^) smaller than wild type, respectively. As previously shown in Figure 1B, *pgm* null cells were 25% smaller at 4.24 µm^2^. Importantly, of the six known *E. coli* enzymes that utilize UDP-glucose (GalE, GalT, Ugd, OpgG, OpgH, and OtsA) mutations in only one, *opgH*::*kan*, led to a statistically significant size reduction (p>0.05). (Length and width data of the various mutants is presented in Table S2.) Why mutations in *galU* and *opgH* do not reduce cell size to the same extent as the *pgm*::*kan* mutation is not readily apparent, however it is reminiscent of what we have observed for mutations in the analogous genes in *B. subtilis* [6].

### OpgH inhibits division in a UDP-glucose-dependent fashion

Based on our study of the parallel pathway in *B. subtilis*, we speculated that OpgH, rather than Pgm or GalU, was the UDP-glucose-dependent regulator of cell division in *E. coli*. To test this hypothesis, we measured the average size of cells expressing either *pgm*, *galU*, and *opgH* (fused with an N-terminal thioredoxin and a C-terminal polyhistidine) from a high-copy plasmid cultured in nutrient-rich media.

Consistent with our prediction, induction of *thio*-*opgH*-*his*, though not *thio*-*pgm*-*his* nor *thio*-*galU*-*his*, led to severe filamentation indicative of a block in cell division (Figure 2C, 2D). Cells expressing *thio*-*opgH*-*his* increased cell length over five-fold, from an average of ∼2.5 µm to ∼13.2 µm after 4 h of induction. Expression of either the *thio*-*pgm*-*his* or *thio*-*galU*-*his* fusion had a negligible impact on cell size.

UDP-glucose was absolutely required for OpgH-mediated division inhibition. Expression of wild-type *thio*-*opgH*-*his* in a *pgm*::*kan* background had no significant impact on cell size (Figure 2C, 2D; Figure S4). Cell size was similarly wild-type in cells expressing a mutant allele of *opgH* (*PIC249AIA*) defective in residues of the putative UDP-glucose binding site [17]. Strains with this mutation were also unable to complement normal glucosyltransferase function (Figure S5). Together, the data presented in Figure 2 suggests OpgH functions as a UDP-glucose-dependent antagonist of cell division.

### OpgH regulates cell size independent from its role in OPG synthesis

OpgH is an inner-membrane glucosyltransferase that synthesizes osmoregulated periplasmic glucans (OPGs); branched glucans consisting of 5 to 13 glucose residues joined by *β*-1–2 linkages and branched by *β*-1–6 linkages [18], [19]. OpgH is responsible for the synthesis and subsequent periplasmic delivery of *β*-1–2 poly-glucose chains from UDP-glucose. *opgH* is co-transcribed with *opgG*, which encodes a periplasmic protein required for generating the *β*-1–6 branches [20].

The loss of OPG synthesis has been implicated in a range of abnormal phenotypes including deficiencies in: envelope stability, flagellar synthesis and motility, phage infectivity, biofilm formation, and pathogenicity [21], [22], [23], [24], [25], [26], [27], [28], [29]. While some of these phenotypes can be attributed to an altered cell envelope, the majority are tied to aberrant activation of the Rcs phosphorelay [22], [30], [31]. To eliminate the possibility that the cell size defect was a secondary consequence of either lacking OPGs and/or Rcs-mediated gene expression, we examined the size of cells defective for OPG synthesis or Rcs activity.

Our data shows that OpgH's role in size modulation is independent of OPG synthesis or activation of the Rcs system. Inactivating *opgG*, thereby eliminating OPG production either by a non-polar mutation in *opgG* or by complementing *opgH* in trans in an *opgGH* double mutant, had no impact on cell size (Figure 2A; Figure S6A).

Based on previous reports indicating that one of the six promoters driving *ftsZ* is positively regulated by Rcs [32], [33], we were particularly concerned that induction of Rcs in the absence of UDP-glucose or OPGs might lead to overexpression of *ftsZ*, which in turn would reduce cell size. However, FtsZ levels are wild-type in Δ*pgm*, Δ*galU*, and Δ*opgH* mutant cells (Figure S6B). (FtsZ would have to be overexpressed by ∼50% to translate into a size reduction of 15–20% [10], [34].) Furthermore, a Δ*opgH*/Δ*rcsB* double mutant was ∼10% smaller than an Δ*rcsB* single mutant (Figure S6B). Deletion of *rpoS*, a transcriptional target of the Rcs system encoding the stationary phase transcription factor Sigma S [35], [36], had no impact on Δ*opgH* or Δ*pgm* mutant size (Figure S6C). This data is not the first example of wild-type FtsZ levels in circumstances of Rcs induction [37], [38].

### OpgH localizes to the division ring only in nutrient-rich conditions, yet independent of UDP-glucose

**Figure 3:**
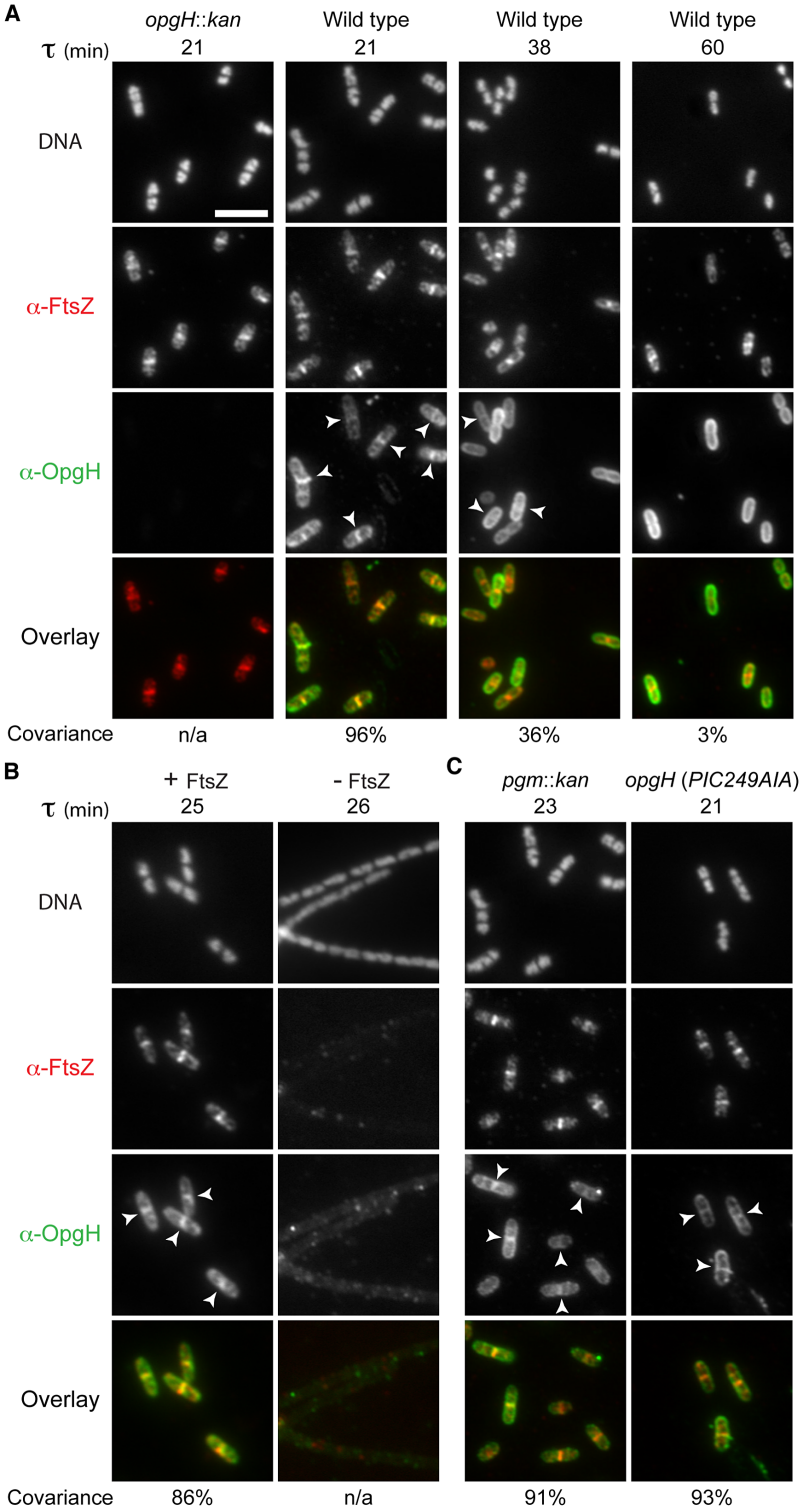
OpgH localizes to midcell in a growth rate- and FtsZ-dependent manner. Immunofluorescence localization of FtsZ and OpgH in various growth conditions or genetic backgrounds. (A) OpgH localizes at midcell with FtsZ only at fast growth rates. Wild type cells were grown in either LB-glucose (τ = 21′), AB-glucose + casamino acids (τ = 38′), or AB-glucose (τ = 60′). (B) OpgH is unable to localize to midcell in the absence of FtsZ. A strain encoding a sodium salicylate inducible copy of *ftsZ* (PL3180) was grown to mid-log phase and back-diluted into LB broth ± inducer (2.5 µM sodium salicylate) for 2.5 h. (C) The frequency of OpgH at midcell is independent of UDP-glucose. Congenic strains either encoding a deletion of a key gene in UDP-glucose biosynthesis (*pgm*::*kan*) or a mutation in OpgH's putative UDP-glucose binding site. (A–C) DNA is stained by DAPI. Bar = 5 µm. White arrowheads indicate OpgH midcell localization. OpgH is in green and FtsZ is in red in the overlays. The percent covariance of FtsZ and OpgH at midcell is indicated below the micrographs.

To determine OpgH's subcellular localization pattern, we used antiserum raised against OpgH's N-terminal cytoplasmic domain. During growth in LB-glucose (τ = 21), OpgH exhibited both peripheral and midcell localization. Of the 203/227 (89%) cells with an FtsZ ring, OpgH colocalized with FtsZ at midcell in 96% (195/203) of cells (Figure 3A). OpgH midcell localization was contingent on nutrient-rich conditions. At a slower growth rate (τ = 38), the covariance of OpgH and FtsZ medial localization was significantly reduced [FtsZ at midcell 69% (170/248); covariance 36% (61/170)]. When growth rates were further reduced by culturing cells in AB-glucose (τ = 60), OpgH was relegated to the cell periphery, rarely colocalizing with an FtsZ ring (3% 3/112) [55% (112/207) of cells had an FtsZ ring]. OpgH localization to midcell was dependent on FtsZ. We were unable to detect an instance in which OpgH localized to midcell in the absence of an FtsZ ring. Further, medial localization of OpgH was abolished when *ftsZ* was depleted (Figure 3B).

Based on our work the *B. subtilis* functional homolog UgtP, we speculated that changes in the intracellular levels of OpgH's substrate, UDP-glucose, might be responsible for its nutrient-dependent localization [6]. However, OpgH localization was unperturbed in either a background unable to synthesize UDP-glucose (*pgm*::*kan*) or a mutation in the putative UDP-glucose binding region (Figure 3C). Midcell covariance of OpgH and FtsZ localization at midcell in the *pgm*::*kan* cells was 91% (178/196) and 93% (212/227) in the nucleotide sugar-binding mutant when cultured in LB-glucose. Together, this data supports a model in which OpgH dynamically localizes to the division machinery in a growth rate-dependent, but UDP-glucose-independent manner.

### The N-terminus of OpgH is both necessary and sufficient for division inhibition

**Figure 4:**
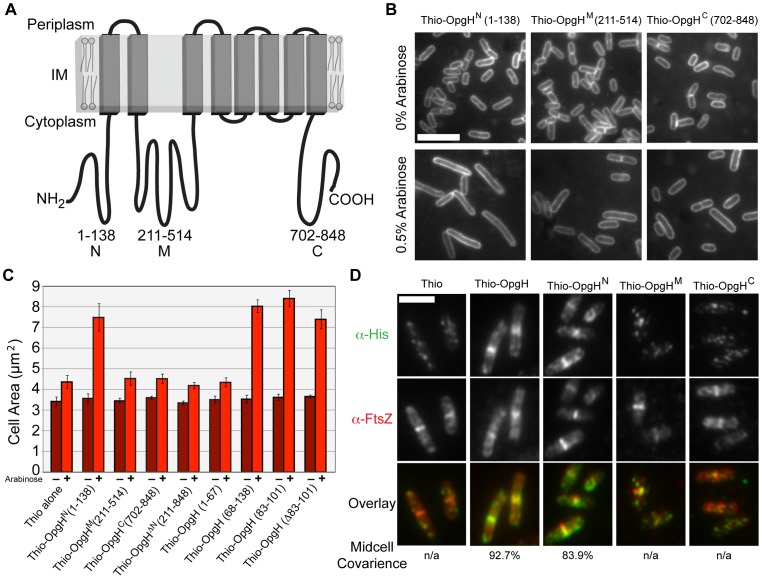
The N-terminal cytoplasmic region of OpgH is necessary and sufficient to inhibit cell division. (A) A schematic representation of the inner-membrane glucosyltransferase OpgH [39]. (B) Overexpression of OpgH^N^ increases cell size in the *opgH*::*kan* strain. Micrographs of cells encoding arabinose inducible N-terminal thioredoxin fusions to each of OpgH's cytoplasmic domains cultured in LB±0.5% arabinose. Cells are stained with FM4-64. Bar = 5 µm. (C) Cell area measurements of cells with the various *thio*-*opgH*-*his* constructs. Cells were cultured in LB with either 0% arabinose (dark red bars) or 0.5% arabinose (light red bars). Error bars equal standard deviation (n = 3). (D) Immunofluorescence micrographs of various *P_ara_*::*thio-opgH-his* constructs following 2 h of induction in LB+0.5% arabinose. Overlays of OpgH (green) and FtsZ (red) localization are on the bottom row. Bar = 3 µm. See also Figure S7 (localization data on additional deletion constructions).

OpgH is an 848 amino acid (97 kDa) integral inner-membrane protein. Reporter fusion analysis indicates that OpgH is composed of eight transmembrane domains with both N- and C-termini residing in the cytoplasm (Figure 4A) [39]. OpgH has three significant cytoplasmic domains referred to here as OpgH^N^ (1–138), OpgH^M^ (211–514), and OpgH^C^ (702–848). OpgH^M^, the largest domain, contains the protein's putative UDP-glucose binding domain based upon sequence homology.

To identify determinants within OpgH required for division inhibition, we expressed thioredoxin/polyhistidine fusions to *opgH^N^*, *opgH^M^*, or *opgH^C^* from an arabinose inducible promoter on multicopy vectors in an *opgH*::*kan* mutant background. Induction of *thio*-*opgH^N^*-*his*, but not *thio*-*opgH^M^*-*his* or *thio*-*opgH^C^*-*his*, resulted in a substantial increase in cell size relative to an uninduced control (Figure 4B, 4C). The area of cells expressing Thio-OpgH^N^-His increased more than two-fold ∼3 h post-induction (from 2.9 µm to 5.9 µm), indicative of a partial block in division. The differential in division inhibition efficacy between OpgH and OpgH^N^ may be the consequence of the former being at the membrane where it can more readily access the division apparatus. Expression of an OpgH^N^ deletion mutant (*thio*-*opgH*(Δ1-210)-*his*) had no impact on cell size (Figure 4C). These data are consistent with a model in which UDP-glucose binding to OpgH^M^ leads to a conformational change that promotes OpgH^N^ mediated division inhibition.

Expression of thioredoxin fusions to a set of nested deletions within the OpgH^N^ domain identified an 18 amino acid peptide (residues 83–101) that was sufficient for division inhibition (Figure 4C; Figure S7). However, systematic alanine substitutions to this region or deletion of all 18 residues had no significant impact on the ability of OpgH^N^ to mediate division inhibition (Figure 4C; data not shown). This finding suggests the presence of at least one additional determinant within OpgH^N^ that is sufficient for its role in division inhibition.

Consistent with a role in cell division, OpgH^N^ localized to midcell independent of the rest of the protein. Immunofluorescence microscopy using antibodies against the His tag, indicated that of the three cytoplasmic domains, only Thio-OpgH^N^-His exhibited medial localization on its own. During growth in LB-glucose, 84% (379/452) of cells expressing *thio*-*opgH^N^*-*his* displayed colocalization with FtsZ at midcell (Figure 4D). Strikingly, a Thioredoxin fusion to the 18 residue fragment of OpgH^N^ (residues 83–101) that was sufficient for division inhibition, also colocalized with FtsZ in 82% (169/205) of cells (Figure S7B).

Although OpgH^N^ was sufficient for medial localization, it was not necessary. A thioredoxin fusion to the OpgH(Δ1–210), the N-terminal deletion mutant, colocalized with FtsZ 67% of the time (148/221) during growth in LB-glucose, implying the N-terminal domain is not necessary for medial localization of OpgH (Figure S7B). Given the inability of the OpgH^M^ or OpgH^C^ domains to localize on their own, we hypothesize that the second localization determinant is situated in a transmembrane and/or periplasmic region of the protein (see Discussion).

### OpgH inhibits division by blocking FtsZ ring formation

In light of our finding that cytoplasmic OpgH^N^ was capable of blocking division in vivo (Figure 4B, 4C), as well as genetic data suggesting that the loss of UDP-glucose positively impacts FtsZ assembly at midcell (Figure 1D), we speculated that OpgH might modulate cell division through direct interactions with FtsZ. To test this possibility, we measured the proportion of cells with FtsZ rings following induction of either *thio-his*, *thio*-*opgH*-*his* or *thio*-*opgH^N^*-*his*. If OpgH inhibits division by antagonizing FtsZ polymerization dynamics, then induction of *thio*-*opgH*-*his* or *thio*-*opgH^N^*-*his* should reduce the number of FtsZ rings.

**Figure 5:**
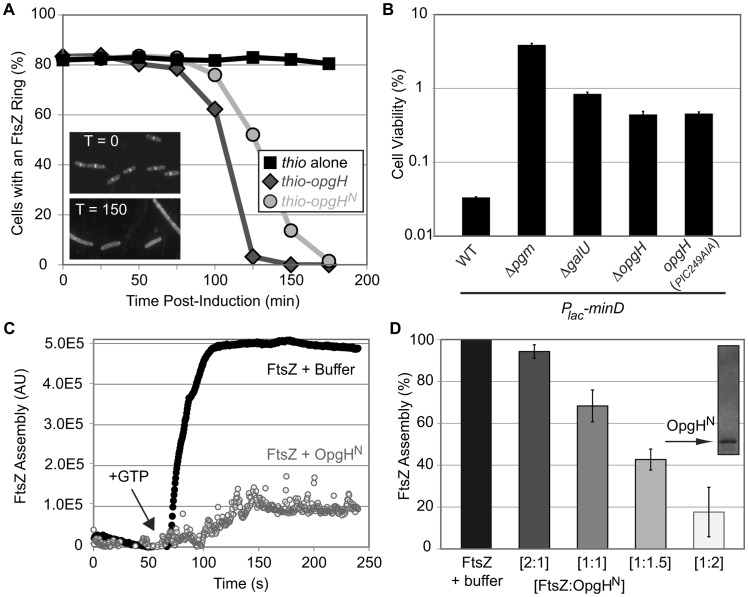
OpgH^N^ is an inhibitor of FtsZ assembly. (A) Induction of OpgH inhibits FtsZ assembly in vivo. Cells were sampled and imaged for immunofluorescence microscopy at 25 minute intervals following the induction of either *thio-his* (black squares), *thio*-*opgH*-*his* (dark grey diamonds), or *thio*-*opgH^N^*-*his* (light grey circles). Cells were cultured in LB. >200 cells were evaluated per sample. Representative α-FtsZ immunofluorescence micrographs from time points 0′ and 150′ are shown in the lower left. (B) Mutations that disrupt synthesis of UDP-glucose or OpgH itself suppress the lethality of MinD overexpression. MinD is overexpressed by ∼2-fold, which is at the threshold of lethality in WT. Error bars equals standard deviation (n = 3). (See Figure S2 for relative MinD expression levels.) (C) A representative 90° angle light scattering plot of FtsZ assembly ± OpgH^N^. FtsZ is at 5 µM, OpgH^N^ is at 10 µM. Arrow indicates addition of 1 mM GTP. (D) Concentration-dependent inhibition of FtsZ polymerization by OpgH^N^. The ratio of FtsZ to OpgH^N^ is listed below. FtsZ is at 5 µM in all cases. (See Figure S9A, S9B for additional controls.)

For this experiment, dilutions were calibrated to ensure cells would be in early exponential growth at each time point. Samples were taken at 25-minute intervals for ∼3 h, fixed, and the percentage of cells with an FtsZ ring were scored using immunofluorescence microscopy. Induction of both *thio*-*opgH*-*his* as well as *thio*-*opgH^N^*-*his* nearly abolished cells with FtsZ rings by ∼2.5 h post-induction (Figure 5A).

As an additional test of the impact of UDP-glucose and OpgH on FtsZ assembly dynamics in vivo, we evaluated the effects of overexpressing the spatial cell division regulator MinD. The precise temporal and spatial regulation of cell division is achieved through a concert of factors that modulate FtsZ assembly. Overexpression of an FtsZ inhibitor, and the associated lethality, can be balanced by deletion of another inhibitor [6], [40].

Consistent with defects in UDP-glucose synthesis or deletion of *opgH* enhancing FtsZ assembly, inactivating genes in the metabolic pathway suppressed lethality associated with *minD* overproduction (Figure 5B). Two-fold overexpression of MinD reduced the plating efficiency of wild-type *E. coli* to 0.03%. However, overexpression of MinD to identical levels in *pgm* null cells resulted in a plating efficiency of 3.9%, >100-fold higher than in the wild-type background (Figure 5B; Figure S2).

Consistent with OpgH interacting directly with FtsZ to delay division, an *opgH*::*kan* mutation increased the viability of cells overexpressing *minD* by ∼1.5 fold (Figure 5B; Figure S2). Cells expressing *opgH*(*PIC249AIA*) phenocopied the *opgH* null results, further corroborating that UDP-glucose binding is essential for OpgH mediated inhibition of FtsZ assembly. In contrast to a loss-of-function mutation in *pgm*, *opgH*::*kan* was unable to suppress the heat-sensitivity of *ftsZ84* allele.

### Genetic data suggests that OpgH reduces the pool of FtsZ available for assembly into the cytokinetic ring

To position OpgH within the regulatory hierarchy responsible for the temporal and spatial control of cell division, we examined the phenotypes of cells defective in either UDP-glucose synthesis (Δ*pgm*) or *opgH* with defects in one of three previously characterized FtsZ antagonists: *minCDE*, *slmA*, or *clpX*. We reasoned that if OpgH shared a role in nucleoid occlusion, ring constriction, or preventing immediate division ring reassembly, then the double knockouts might exacerbate defects in division rings bisecting nucleoids, augmented cell size, or increased rate of minicells.

None of the double mutants had defects in growth rate, increased rates of division rings over unsegregated nucleoids, or a higher rate of inaccurately placed FtsZ rings during growth in LB-glucose (Table S1, data not shown). However, supporting a model in which OpgH reduces the effective concentration of FtsZ, a loss-of-function mutation in either *opgH* or *pgm* reduced the length of the abnormally long *minCDE* null mutants by approximately half (Figure S8; Table S1). This finding suggests that the pools of FtsZ available for assembly into the cytokinetic ring are elevated in the absence of either *opgH* or *pgm*.

### OpgH^N^ interacts directly with FtsZ to inhibit assembly

Based on the genetic evidence suggesting that OpgH antagonizes division through direct interactions with FtsZ, we next determined if purified OpgH^N^ was sufficient to inhibit FtsZ assembly in vitro. For this experiment we employed 90° angle light scattering, a functional assay for FtsZ assembly [41], [42]. All experiments were performed with OpgH^N^ (residues 1–138) and *E. coli* FtsZ in their native (untagged) form.

Consistent with our genetic data, OpgH^N^ inhibited FtsZ assembly in a dose-dependent fashion (Figure 5C, 5D). At a 1∶1 ratio of FtsZ to OpgH^N^, FtsZ assembly was reduced by more than 30%. At higher ratios of FtsZ:OpgH^N^, 1∶1.5 and 1∶2, FtsZ assembly was reduced further by ∼60% and ∼80%, respectively. Heat-inactivating OpgH abolished its ability to inhibit FtsZ assembly (Figure S9A, S9B). This level of inhibition is slightly less potent than other *E. coli* FtsZ inhibitors [43], [44], [45], [46], however it is consistent with a model in which OpgH functions to delay division, not prevent it.

### OpgH raises the apparent critical concentration of FtsZ required for GTP hydrolysis

Although 90° angle light scattering is valuable for measuring gross FtsZ assembly, it does not provide insight into the particular mechanism by which FtsZ assembly is obstructed. To elucidate the mechanism by which OpgH inhibits FtsZ assembly, we next determined the impact of OpgH^N^ on FtsZ's intrinsic GTPase activity. FtsZ binds to GTP as a monomer, however dimerization is required for the formation of the GTPase active site and GTP hydrolysis (for reviews of FtsZ assembly dynamics see [7], [47], [48], [49], [50]). Factors that bind to FtsZ monomers and prevent them from being added to growing polymers (so-called sequesters) reduce GTP hydrolysis activity by interfering with dimerization and the formation of the GTPase active site [7], [51], [52], [53]. Conversely, factors that inhibit FtsZ assembly through other means (severing preformed polymers or interfering with lateral interactions between polymers) have little impact on GTP hydrolysis [43], [54], [55], [56].

**Figure 6:**
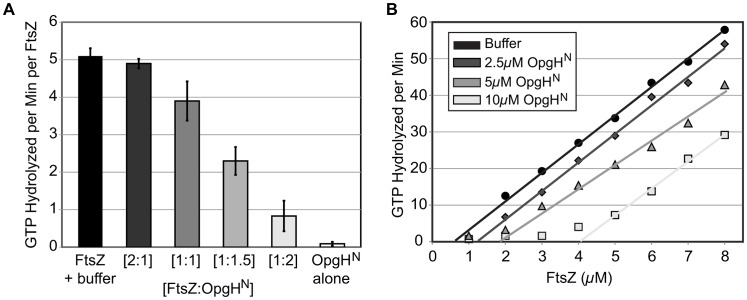
OpgH^N^ appears to function as an FtsZ monomer sequestering protein. (A) Concentration-dependent inhibition of FtsZ's GTPase activity by OpgH^N^. The GTP hydrolysis rate of 5 µM FtsZ is shown at differing ratios of OpgH^N^. OpgH^N^ alone is at 5 µM. Error bars equal standard deviation (n = 3). (B) FtsZ GTPase rates at increasing concentrations of OpgH^N^. The critical concentration for assembly of FtsZ was determined at OpgH^N^ concentrations of 0 µM, 2.5 µM, 5 µM, or 10 µM. (See Figure S9C, S9D for additional controls.)

Consistent with a mechanism in which OpgH sequesters FtsZ monomers and prevents them from assembling into single-stranded polymers, OpgH^N^ reduced FtsZ's intrinsic GTP hydrolysis activity in a dose-dependent manner. Under our conditions, FtsZ hydrolyzed GTP at a rate of 5.1 GTP/minute/FtsZ (Figure 6A). GTP hydrolysis was reduced by 25% at a 1∶1 ratio (FtsZ:OpgH^N^), 55% at 1∶1.5, and 84% at 1∶2. OpgH^N^ did not exhibit any significant GTPase activity on its own. Heat-treated OpgH^N^ had no effect on FtsZ's GTPase activity (Figure S9C). Further, control experiments using a different GTPase, demonstrate that OpgH^N^ does not interfere with the regenerative, NADH coupled GTPase assay (Figure S9D).

Monomer binding reduces the pool of FtsZ subunits available for assembly. A hallmark of a sequestration-like mechanism is an increase in the apparent critical concentration (CcApp) of FtsZ required for GTP hydrolysis. To determine if OpgH^N^ impacts FtsZ's CcApp, we measured GTP hydrolysis rates in reactions that kept levels of OpgH^N^ constant, but increased the FtsZ concentration. Plotting GTP hydrolysis per minute versus FtsZ concentration identifies the X intercept for each OpgH^N^ concentration. This intercept is equivalent to FtsZ's critical concentration under that condition [53].

In support of a model in which OpgH is a monomer binding protein, the addition of OpgH^N^ increased FtsZ's CcApp for GTP hydrolysis. In the absence of OpgH^N^, *E. coli* FtsZ exhibited a CcApp of 0.69 µM. The CcApp was increased to 1.31 µM at a 2.5 µM OpgH^N^ concentration, to 1.85 µM at 5 µM OpgH^N^, and finally to 4.08 µM in 10 µM OpgH^N^ (Figure 6B).

## Discussion

**Figure 7:**
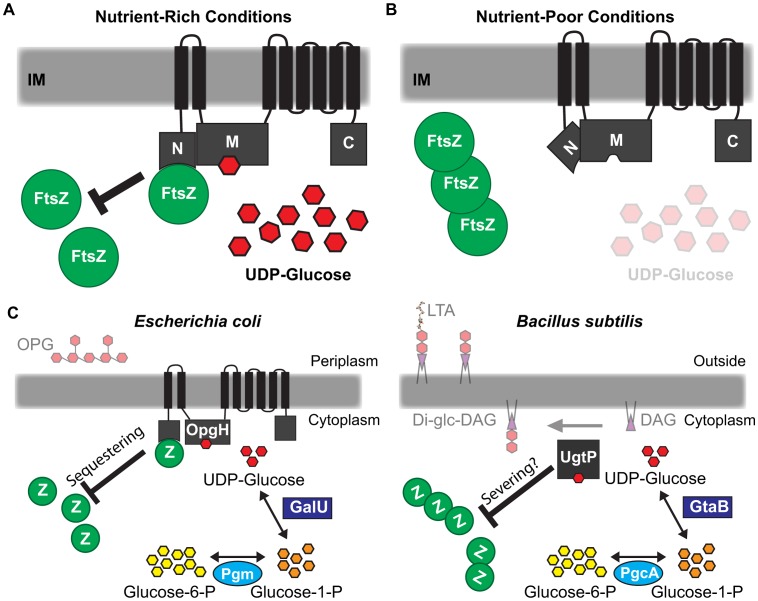
Glucosyltransferase OpgH couples cell size to nutritional availability and growth rate in *E. coli*. (A) The nucleotide sugar UDP-glucose acts as a proxy for nutritional status to ensure cells maintain an optimal size for a given growth rate. OpgH blocks division by inhibiting assembly of the essential bacterial cytoskeleton protein FtsZ. OpgH bound to UDP-glucose assumes a conformation where the N-terminus is able to interact with FtsZ. This interaction effectively reduces the pool of FtsZ able to participate in the formation and maturation of the FtsZ ring. (B) In nutrient-poor conditions, UDP-glucose is less available and no longer serves to promote interaction between OpgH and FtsZ. Consequently, division is unobstructed and cell size does not increase. (C) Evolutionarily divergent organisms *E. coli* and *B. subtilis* both utilize UDP-glucose and unrelated glucosyltransferases to coordinate growth rate-dependent size homeostasis. Both organisms have co-opted sugar transferases activated by UDP-glucose to antagonize assembly of FtsZ by different mechanisms.

Classic work conducted over 50 years ago first identified nutritional content and growth rate as primary determinants of bacterial cell size [2]. Here we report that two factors, UDP-glucose and the glucosyltransferase OpgH, govern the regulatory circuit responsible for coordinating *E. coli* cell size with nutrient availability. Our genetic and biochemical data support a model in which binding of UDP-glucose by OpgH promotes a conformational change revealing an FtsZ interaction site on the OpgH^N^ domain. Interactions between OpgH^N^ and FtsZ obstruct assembly and/or maturation of the cytokinetic ring, delaying division and increasing cell size (Figure 7A). During slow growth in nutrient-poor conditions or defective for UDP-glucose production, OpgH assumes a conformation that obscures the interaction between OpgH^N^ and FtsZ. FtsZ assembly is able to proceed unimpeded, reducing cell size (Figure 7B). OpgH, specifically OpgH^N^, is well conserved in the order Enterobacteriales, suggesting functional conservation in close relatives of *E. coli* (Figure S10).

Based on our genetic and biochemical data, we propose that nutrient-dependent activation of OpgH leads to a reduction of FtsZ available for assembly into the cytokinetic ring. FtsZ levels are constant regardless of growth rate [57]. Nutrient-dependent increases in OpgH activity should translate into proportional reductions in the pool of available FtsZ subunits. Cells increase in size until they have accumulated sufficient FtsZ to overcome OpgH-mediated inhibition and support assembly of a mature cytokinetic ring [1]. This model is consistent with data from the Vicente lab demonstrating that modest reductions in FtsZ pools lead to a transient increases in cell size, but do not impact the timing of division under steady-state conditions [58].

### Multiple determinants ensure OpgH localization to the division machinery

OpgH's localization to the division apparatus only occurs in nutrient-rich conditions (Figure 3). Intriguingly, this growth rate-dependent localization is not controlled by interaction with its substrate, UDP-glucose, unlike its functional *B. subtilis* homolog UgtP [6]. Thus, the mechanism behind OpgH's dynamic nutrient-dependent localization remains to be elucidated.

Analysis of OpgH deletion constructs suggests the presence of at least two determinants within the OpgH polypeptide are required for localization to the cytokinetic ring (Figure 4D; Figure S7B). One such determinant is within the soluble OpgH^N^ domain. The other determinant resides somewhere in residues 211–848. Intriguingly, residues 651–713, encoding OpgH's 7^th^ and 8^th^ transmembrane domains, share homology to the SEDS (shape, elongation, division, and sporulation) family of proteins. The essential division protein FtsW and the shape determining protein RodA, two well-studied *E. coli* SEDS proteins, localize to the division apparatus through FtsZ-independent interactions [59], [60]. We hypothesize that OpgH employs its SEDS-like domain to bolster its medial localization by interacting with other components of the division machinery.

Specifically why OpgH employs more than one mechanism for localization to the cytokinetic ring is not immediately apparent. However, we speculate that concentrating OpgH at midcell may be essential to ensure that its effective concentration is high enough to meaningfully impact cell division. Although we were unable to obtain quantitative measurements of native OpgH levels, similar to other groups [39], qPCR data suggests *opgH* transcripts are ∼1000-fold less abundant than *ftsZ* transcripts (data not shown). A localization mechanism that depends on factors that localize to the division site downstream of FtsZ, would ensure that OpgH remains at midcell during the duration of the Z-period and is not displaced by competition with other FtsZ binding proteins.

### A remarkable example of convergent evolution

When viewed in the context of the parallel pathway in *B. subtilis*, this study highlights an exceptional example of convergent evolution. *E. coli* and *B. subtilis*, organisms separated by a greater evolutionary distance than *Homo sapiens* and *Saccharomyces cerevisiae*, both utilize UDP-glucose and unrelated glucosyltransferases in the regulatory circuit coupling cell size with growth rate and nutrient availability (Figure 7C).

Why UDP-glucose-utilizing glucosyltransferase enzymes were chosen to coordinate this phenomenon is an intriguing question. In general, moonlighting enzymes tend to be more valuable if the multiple functions are both beneficial at the same time [61]. Thus, the question arises: Aside from growth in rich nutrient conditions, are there circumstances in which an increased need for their sugar transferase activity coincides with a need to prevent division?

Both effectors have previously described roles in envelope biogenesis: OpgH produces periplasmic glucans and UgtP synthesizes the Di-glc-DAG anchor for LTA. These products are particularly important during conditions that prompt the osmotic stress response [8], [62], [63]. Curiously, shifting the osmotic or turgor pressure has been reported to cause a temporary arrest of cell division that is alleviated only after cells equilibrate to the new conditions [64], [65]. Thus, while our work describes a role for OpgH and UgtP in nutrient-dependent size control, we speculate that both proteins may also function to inhibit division during osmotic stress or other cell envelope perturbations.

UDP-glucose is an ideal signaling molecule for transmitting information to the division apparatus under both conditions of rapid growth and cell envelope stress. Its accumulation is directly coupled to central carbon metabolism and is likely to accumulate primarily under nutrient-rich conditions, the same conditions that support multifork replication and necessitate an increase in cell size. Likewise, consistent with a role in the osmotic stress response pathway, genes involved in UDP-glucose synthesis are up-regulated during cell envelope stress and UDP-glucose is directly incorporated into the cell envelope [66], [67].

Curiously, synthesis of both OPG and LTA is the primary source of diacylglycerol in *E. coli* and *B. subtilis* [68], raising the possibility that diacylglycerol may serve as a secondary messenger in the regulatory circuit governing bacterial cell size. (FabH, an enzyme involved in key steps of fatty acid biosynthesis, has recently been implicated in the nutrient-dependent control of cell size, suggesting another potential role for lipids in cell size homeostasis [69].)

In closing, our findings represent a major advance in our understanding of cell size control in *E. coli*, the predominant model system for the study of bacterial physiology. Moreover, through comparison with a parallel pathway in *B. subtilis*, our work reveals conserved aspects of growth rate regulation and cell size control – including conservation of UDP-glucose, a molecule common to all domains of life, as a proxy for nutrient availability and the use of moonlighting enzymes to couple growth rate-dependent phenomena to central metabolism – that are likely to be broadly applicable.

## Materials and Methods

### Strains and media

*E. coli* strains and plasmids and their construction are described in Supporting Information (Text S1, Table S3, and Table S4). Cells were cultured in Luria-Bertani (LB) ±0.2% glucose or AB defined media [70] supplemented with 10 µg/ml thymine, ±0.5% casamino acids (CAA), and either 0.2% glucose or 0.4% succinate as a carbon source. Cells were grown at 37°C and used for experimentation at early exponential growth phase (an OD of 0.15–0.3) unless otherwise stated. Standard techniques were employed for cloning, P1vir transductions, and other genetic manipulations.

### Quantitative immunoblotting

Cells were lysed at early to mid-log phase (an OD_600_ of 0.2–0.5) using physical or chemical techniques. Lysates were then normalized to either OD or total protein using a bicinchoninic acid (BCA) assay and subjected to SDS-PAGE. Immunoblots were performed using either rabbit α-MinD antibody (the gift of William Margolin), chicken α-His antibody (Millipore), rabbit α-FtsZ (the gift of David Weiss), or rabbit α-DnaA (the gift of Jon Kaguni) with cognate goat α-rabbit or donkey α-chicken secondary antibody conjugated to horseradish peroxidase (Jackson Immunoresearch). Band intensity was determined using ImageJ software and processed in Microsoft Excel [71].

### Immunofluorescence microscopy

Microscopy performed was essentially similar to [10]. Cells were fixed in paraformaldehyde similar to as described in [72]. His-tagged proteins were detected using a chicken α-polyhistidine tag antibody (Millipore) with cognate goat α-chicken serum conjugated to Alexa488 (Invitrogen). FtsZ was detected using affinity-purified polyclonal rabbit α-FtsZ serum (the gift of William Margolin) in combination with goat α-rabbit serum conjugated to Alexa546 (Invitrogen). OpgH was detected using mouse α-OpgH^N^ polyclonal serum with goat anti-mouse Alexa488 (Invitrogen). Genetic content was stained with DAPI.

### Cell size measurements

Cells in early to mid-log phase were stained with FM-464 and adhered to 15-well glass slides using poly-L-lysine. The length and width of cells were calculated using the Openlab software. Only length was measured in cases of severe cell filamentation when width was irrelevant to the interpretation.

### *ftsZ84* and *minD* plating efficiency

Plating efficiency was done as previously described [73]. Briefly, strains encoding *ftsZ84* or an inducible copy of *minD* were cultured in permissive conditions to early/mid-log phase (OD_600_ 0.1–0.4), back-diluted to an OD_600_ of 0.01 and grown to an OD_600_ of 0.15–0.3. Cultures were normalized to optical density and then serially diluted from 10^−1^ to 10^−8^. Equal volumes were plated at permissive conditions (LB with 0.05% NaCl at 30°C for *ftsZ84*; LB-glucose + 0 mM IPTG for *minD*) and restrictive conditions (LB with 0.05% NaCl at 42°C for *ftsZ84*; LB-glucose + 0.15 mM IPTG for *minD*). Plating efficiency was calculated as the ratio of colony forming units at restrictive versus permissive conditions.

### Protein purification

Untagged *E. coli* FtsZ was purified as described previously [42]. Native OpgH^N^ was purified using the IMPACT Protein Purification System (New England Biolabs) as follows. BL21(DE3) cells harboring pBH616 (pTYB4 + *opgH^N^*) was grown in one-liter cultures with 100 µg/ml ampicillin at 37°C to mid-log, then induced with 1 mM IPTG for 4–6 h. (Inducting at temperatures under 37°C did not yield functional OpgH^N^.) Cells were pelleted, washed in 1×PBS (pH 7.4), repelleted, and frozen at **−**80°C for later use. On the day of purification, cell pellets were thawed and resuspended in 30 ml of ice-cold IMPACT lysis buffer (20 mM Tris-HCl, 500 mM NaCl, 1 mM EDTA, pH 8) containing 1 mM 4-(2-aminoethyl)benzenesulfonyl fluoride hydrochloride (Sigma). Multiple passes in a prechilled French press cell at 1,000 lb/in^2^ lysed cells. All subsequent steps were performed at 4°C. Lysates were cleared by centrifugation, then loaded on to pre-equilibrated columns with 10–15 ml of chitin beads (New England Biolabs). The column was then washed with ∼10 column volumes of IMPACT lysis buffer. Subsequently, IMPACT lysis buffer containing 0.05 M dithiothreitol (DTT) was loaded to stimulate Intein auto-cleavage. The column was left incubating with DTT at 4°C overnight (16–20 h). The eluate yielded both the OpgH^N^-Intein fusion (71.9 kDa) and OpgH^N^ (15.9 kDa). The fractions were pooled and transferred into dialysis tubing (7,000 MWCO) and concentrated from ∼25 ml to ∼1 ml using polyethylene glycol (PEG) 12,000. The 1 mL was immediately loaded onto a S-300 size exclusion column and equilibrated in OpgH^N^ buffer (20 mM Tris-HCl, 100 mM NaCl, 10% glycerol, pH 8). OpgH^N^ fractions were pooled and transferred to dialysis tubing (7,000 MWCO) and concentrated with PEG. Aliquots were flash frozen in liquid nitrogen and stored at **−**80°C. Protein concentrations were determined by a Coomassie Plus (Pierce) assay using a SPECTRAmax Plus spectrophotometer (Molecular Devices) using a BSA standard.

### 90° angle light scattering assay

Light scattering assays were performed as described previously using a DM-45 spectrofluorimeter (Olis) [40], [73]. Readings were taken every 0.5 s at 30°C. A baseline was established ∼60 s previous to the addition of 1 mM GTP. The reaction mixtures contained a final concentration of 5 µM FtsZ diluted in polymerization buffer (50 mM morpholineethanesulfonic acid (MES), 2.5 mM MgCl_2_, 1 mM EGTA, 50 mM KCl, pH 6.5) ± OpgH^N^ or OpgH^N^ buffer (20 mM Tris-HCl, 100 mM NaCl, 10% glycerol, pH 8). Data was collected by SpectralWorks (Olis) and exported into Microsoft Excel for processing. Baseline corrections were applied in Microsoft Excel to remove the background signal from unassembled FtsZ.

### Regenerative coupled GTPase assay

FtsZ's GTPase activity was evaluated as previously described [42] using a continuous, regenerative coupled GTPase assay [74]. Briefly, experiments were done in the same buffer conditions as used for light scattering. A 150 µl reaction volume included: 1 mM phosphoenolpyruvate (PEP), 80 units/ml lactose dehydrogenase, 80 units/ml pyruvate kinase, 250 µM NADH, 1 mM GTP then varying amounts of FtsZ and OpgH^N^ or equivalent volume of respective buffer. Absorbance at 340 nm was measured at 30°C for 3 min in a quartz cuvette (1 cm path length) using a SPECTRAmax Plus spectrophotometer (Molecular Devices). Raw data from 60–120 s was translated into activity with the extinction coefficient for NADH at 340 nm of 6220 M**^−^** ^1^ cm**^−^** ^1^.

## Supporting Information

Figure S1 FtsZ84 levels are not elevated in *pgm* null strain. A representative quantitative immunoblot of FtsZ84 levels for WT and *pgm*::*kan* strains encoding the *ftsZ84* allele at both permissive (30°C) and restrictive (42°C) growth conditions (See Figure 1D). The replication protein DnaA is shown as a loading control (LC). Relative expression (below) was calculated using WT grown at 30°C as the reference (n = 3).
(EPS)

Figure S2 MinD overexpression and FtsZ levels are congruent in wild type and UDP-glucose pathway mutants. A representative quantitative immunoblot of MinD overexpression and FtsZ levels in WT and the UDP-glucose pathway mutants encoding a plasmid with *P_lac_*::*minD*. Cells were cultured in LB±0.15 mM IPTG for ∼3 h. Average fold overexpression level displayed below (n = 3).
(EPS)

Figure S3 The cell size defect is confined to the UDP-glucose biosynthesis pathway. Cell area measurements of loss-of-functions mutations in genes adjacent to the UDP-glucose (see Figure 1A). Cells were grown in LB-glucose. >250 cells per strain were evaluated per replicate. Error bar equals standard deviation (n = 3).
(EPS)

Figure S4 Thio-OpgH-His expressed to similar levels. Expression levels for wild-type Thio-OpgH-His, a putative UDP-glucose binding mutant (PIC249AIA), or wild-type OpgH in a *pgm*::*kan* background. Cells were grown in LB±0.5% arabinose.
(EPS)

Figure S5 *opgH*-*gfp* and *thio*-*opgH*-*his* fusion constructs complement for size and glucosyltransferase activity. (A) Cell area distribution of WT (black), *opgH*::*kan* (white), *opgH*::*kan* + *P_lac_*::*opgH*-*gfp* (green), and *opgH*::*kan* + *P_ara_*::*thio*-*opgH*-*his* (red) grown in LB-glucose. The *P_lac_*::*opgH*-*gfp* is induced with 0.08 mM IPTG, while *P_ara_*::*thio*-*opgH* is induced with 0.25% arabinose. >250 cells were evaluated. Averages shown in the inset. (B) Swarm phenotypes are a proxy for glucosyltransferase activity (see Text S1). Expression of either *P_lac_*::*opgH*-*gfp* (in 0.08 mM IPTG) or *P_ara_*::*thio*-*opgH*-*his* (0.25% arabinose) in an *opgH* null led to wild-type swarming. Similar expression using a *opgH* sugar-binding mutant (*) or inactivating UDP-glucose biosynthesis (*pgm*::*kan*) was unable to complement normal activity. Average diameter of swarm distance shown below (n = 3).
(EPS)

Figure S6 The Δ*opgH* cell size defect is independent of osmoregulated periplasmic glucans and Rcs activation. (A) Cell area measurements of an *opgGH* null complemented with either *P_lac_*::*opgG*-*gfp* or *P_lac_*::*opgH*-*gfp*. Cells grown in LB-glucose with 0.1 mM IPTG. (B) A representative quantitative immunoblot of FtsZ levels for WT and the UDP-glucose null strains. Lysates were normalized to total protein using a BSA assay. Relative expression (below) was calculated using WT as the reference (n = 3). (C) Loss-of-function mutations in the UDP-glucose pathway genes combined with null mutations in either the Rcs response regulator *rcsB* (left) or the alternative sigma factor *rpoS* (right). >250 cells per strain were evaluated three times. Error bars equals standard deviation (n = 3).
(EPS)

Figure S7 An 18-amino acid peptide of OpgH is sufficient, though unnecessary, to inhibit division and localize to the division ring. (A) Cell area measurements of *P_ara_*::*thio*-*opgH^N^*-*his* deletion constructs in a *opgH*::*kan* background cultured in LB±0.5% arabinose. Cells were cultured with inducer for ∼3 h. >250 cells were assessed for cell area in each replicate. (B) Immunofluorescence of Thio-OpgH(83–101)-His and Thio-OpgH(211–848)-His after being cultured in LB+0.5% arabinose for 2 h. OpgH localization using an α-His antibody is shown in the top panel, α-FtsZ in the middle panel, with the colocalization displayed on the bottom panel (OpgH in green, FtsZ in red). The covariance of OpgH/FtsZ midcell localization is denoted below. Bar = 3 µm. (C) Quantitative immunoblot of the various Thio-OpgH-His constructs in the *opgH* null cultured in LB±0.5% arabinose. FtsZ is used as a loading control (below).
(EPS)

Figure S8 UDP-glucose pathway mutants reduce filamentation of Δ*minCDE*. *minCDE*::*kan*, Δ*pgm minCDE*::*kan*, Δ*opgH minCDE*::*kan* strains grown in LB-glucose, stained with FM4-64. The average area is listed below (>150 cells counted, n = 3). Bar = 5 µm. A representative immunoblot of FtsZ levels of the mutants is shown below.
(EPS)

Figure S9 Heat-treated OpgH^N^ loses ability to inhibit FtsZ. (A) A representative 90° angle light scattering plot and (B) percent assembly of FtsZ assembly ± heat-treated OpgH^N^. Arrow indicates addition of 1 mM GTP. (C) The rate of GTP hydrolysis of FtsZ ± heat-treated OpgH. (A–C) FtsZ is at 5 µM, heat-treated OpgH^N^ is at 10 µM. OpgH^N^ was subjected to 30 minutes at 90°C to act as the heat-treated control. (D) The GTPase activity of calf intestinal phosphatase (CIP) was measured with either OpgH^N^ buffer (black) or 10 µM OpgH^N^ (blue). CIP's ability is to hydrolyze GTP is unaltered with the addition of OpgH^N^. This demonstrates that OpgH^N^'s activity is specific to FtsZ and is not inhibiting a different reaction in the regenerative NADH-coupled assay. Error bars equal standard deviation (n = 3).
(EPS)

Figure S10 Phylogenetic tree and alignment of the FtsZ inhibiting domain of OpgH. (A) A phylogenetic tree comparing the C-terminus of OpgH^N^ (66–138) with other gammaproteobacteria. The percent identity and percent similarity are listed in parentheses. (B) The protein alignment of OpgH^N^ (66–138) with various other gammaproteobacteria that encode an OpgH homolog. The black box indicates the 18 amino acid region that is sufficient to block division in vivo.
(EPS)

Table S1 Phenotypes of combining defects in UDP-glucose synthesis (Δ*pgm*) or Δ*opgH* with inactivating characterized *E. coli* division inhibitors.
(DOCX)

Table S2 Detailed cell size measurements of mutants associated with the UDP-glucose synthesis.
(DOC)

Table S3 Bacterial strains used in this study.
(DOC)

Table S4 Bacterial plasmids used in this study.
(DOC)

Text S1 A description of media conditions, strain construction, and auxiliary methods.
(DOC)
