# Supplementary material for: A Moonlighting Enzyme Links Escherichia coli Cell Size with Central Metabolism
Source: PLoS Genet. 2013 Jul 25;9(7):e1003663. doi: 10.1371/journal.pgen.1003663 (PMC3723540; doi:10.1371/journal.pgen.1003663)
Supplement: Table S1 — Phenotypes of combining defects in UDP-glucose synthesis (Δpgm) or ΔopgH with inactivating characterized E. coli division inhibitors. (DOCX) [file pgen.1003663.s011.docx]

**Table S1.** Phenotypes of combining defects in UDP-glucose synthesis (Δ*pgm*) or Δ*opgH* with inactivating characterized *E. coli* division inhibitors.

| **Genotype** | **Growth rate (min)** | **Cell area (µm^2^)** | **Rings over nucleoids** |
| --- | --- | --- | --- |
| ***wt*** | 21.2 | 5.66 | 19.9% (130/654) |
| **Δ*pgm*** | 22.7 | 4.28 | 37.2% (246/662) |
| **Δ*opgH*** | 21.2 | 5.03 | 32.1% (212/660) |
| **Δ*minCDE*** | 24.0 | 10.2 | 23.5% (50/213) |
| **Δ*minCDE* Δ*pgm*** | 22.7 | 5.50 | 21.1% (114/540) |
| **Δ*minCDE* Δ*opgH*** | 22.7 | 5.72 | 22.9% (120/524) |
| **Δ*slmA*** | 21.9 | 5.12 | 25.6% (160/632) |
| **Δ*slmA* Δ*pgm*** | 23.0 | 4.00 | 37.3% (208/558) |
| **Δ*slmA* Δ*opgH*** | 21.8 | 4.64 | 31.4% (244/776) |
| **Δ*clpX*** | 21.4 | 5.44 | 18.1% (104/576) |
| **Δ*clpX* Δ*pgm*** | 22.6 | 4.39 | 35.4% (216/710) |
| **Δ*clpX* Δ*opgH*** | 21.8 | 4.72 | 30.0% (212/706) |
