# Supplementary material for: A Moonlighting Enzyme Links Escherichia coli Cell Size with Central Metabolism
Source: PLoS Genet. 2013 Jul 25;9(7):e1003663. doi: 10.1371/journal.pgen.1003663 (PMC3723540; doi:10.1371/journal.pgen.1003663)
Supplement: Table S2 — Detailed cell size measurements of mutants associated with the UDP-glucose synthesis. (DOC) [file pgen.1003663.s012.doc]

**Table S2.** Detailed cell size measurements of mutants associated with UDP-glucose synthesis or utilization cultured in LB-glucose.

| **Genotype** | **Growth rate^a^** | **Area (μm^2^)** | **Length (μm)** | **Width (μm)** | ***oriC*/cell^b^** |
| --- | --- | --- | --- | --- | --- |
| ***wt*** | 21.2/78.3 | 5.66 ± 0.13 | 4.55 ± 0.10 | 1.22 ± 0.03 | 10.2 ± 0.38 |
| **Δ*pgm*** | 22.7/82.1 | 4.24 ± 0.27 | 3.15 ± 0.28 | 1.40 ± 0.01 | 4.33 ± .09 |
| **Δ*galU*** | 22.2/81.0 | 4.66 ± 0.19 | 3.94 ± 0.07 | 1.13 ± 0.03 | 4.76 ± .10 |
| **Δ*galE*** | 21.8/78.9 | 5.53 ± 0.25 | 4.41 ± 0.16 | 1.24 ± 0.01 | 10.2 ± 0.33 |
| **Δ*galT*** | 21.1/79.3 | 5.70 ± 0.27 | 4.43 ± 0.23 | 1.27 ± 0.11 | 10.2 ± 0.42 |
| **Δ*ugd*** | 21.5/79.0 | 5.72 ± 0.27 | 4.53 ± 0.21 | 1.25 ± 0.03 | 9.54 ± 0.35 |
| **Δ*otsA*** | 21.5/78.8 | 5.44 ± 0.27 | 4.33 ± 0.20 | 1.23 ± 0.04 | 10.1 ± 0.42 |
| **Δ*opgG*** | 21.7/78.2 | 5.61 ± 0.30 | 4.50 ± 0.17 | 1.24 ± 0.01 | 10.4 ± 0.35 |
| **Δ*opgH*** | 21.2/78.6 | 5.01 ± 0.06 | 3.7 ± 0.03 | 1.36 ± 0.03 | 6.14 ± 0.27 |
| ***opgH* (*PIC249AIA*)** | 21.4/78.9 | 5.02 ± 0.06 | 3.75 ± 0.05 | 1.34 ± 0.02 | n/a |
| **Δ*pgm* Δ*opgH*** | 22.9/ n/a | 4.34 ± 0.25 | 3.07 ± 0.13 | 1.42 ± 0.03 | n/a |
| **Δ*pgi*** | 21.5/n/a | 5.64 ± 0.04 | 4.54 ± 0.01 | 1.24 ± 0.01 | n/a |
| **Δ*zwf*** | 21.6/n/a | 5.90 ± 0.15 | 4.56 ± 0.11 | 1.29 ± 0.01 | n/a |
| **Δ*rfbA*** | n/a | 6.03 ± 0.08 | 4.89 ± 0.13 | 1.23 ± 0.02 | n/a |
| **Δ*rfbB*** | n/a | 5.75 ± 0.12 | 4.65 ± 0.13 | 1.24 ± 0.02 | n/a |
| **Δ*glgB*** | n/a | 5.54 ± 0.08 | 4.49 ± 0.04 | 1.23 ± 0.02 | n/a |
| **Δ*glgC*** | n/a | 5.43 ± 0.31 | 4.43 ± 0.16 | 1.23 ± 0.03 | n/a |
| **Δ*galF*** | 21.8/n/a | 5.58 ± 0.44 | 4.53 ± 0.25 | 1.23 ± 0.04 | n/a |

The average is presented ± SD (n = 3).

**^a^** Growth rate for the strains cultured in LB-glucose/AB-succinate. The rate is listed in minutes during the exponential period of growth.

**^b^** Cell size is coupled to chromosomal replication in *E. coli*. A reduction in chromosomal origins (*oriC*) per cell indicates a reduction in cell mass. It is shown here to demonstrate the *pgm*, *galU*, and *opgH* deletions have significant *oriC*/cell reductions in agreement with the size measurements.
