# Supplementary material for: A Moonlighting Enzyme Links Escherichia coli Cell Size with Central Metabolism
Source: PLoS Genet. 2013 Jul 25;9(7):e1003663. doi: 10.1371/journal.pgen.1003663 (PMC3723540; doi:10.1371/journal.pgen.1003663)
Supplement: Table S3 — Bacterial strains used in this study. (DOC) [file pgen.1003663.s013.doc]

**Table S3.** Bacterial strains used in this study.

| **Annotation** | **Relevant genotype^a^** | **Source^b^** |
| --- | --- | --- |
| BH140 (MG1655) | *F^-^ λ^-^ ilvG- rfb-50 rph-1* | [1] |
| BH121  (W3110) | *F^-^ λ^-^ rph-1 INV*(*rrnD, rrnE*) | [2] |
| PL1679  (BL21(DE3)) | *F^–^ ompT gal dcm lon hsdS_B_* λ(DE3) | [3] |
| PL2432  (BW25113) | *F^-^ λ^-^ rph-1* ∆*(araD-araB)* ∆*lacZ* ∆*hsdR* ∆*(rhaD-rhaB)* | [4] |
| BH141 | MG1655 Δ*pgm*::*kan* | [4] |
| PL2449 | MG1655 Δ*galU*::*kan* | [4] |
| BH167 | MG1655 Δ*galT*::*kan* | [4] |
| BH169 | MG1655 Δ*otsA*::*kan* | [4] |
| BH171 | MG1655 Δ*galE*::*kan* | [4] |
| BH177 | MG1655 Δ*ugd*::*kan* | [4] |
| BH643 | MG1655 Δ*opgG*::*frt* | Recombineering |
| PL2450 | MG1655 Δ*opgG*::*kan* | [4] |
| PL2455 | MG1655 Δ*opgH*::*kan* | [4] |
| PL2325 | W3110 Δ*pgi*::*kan* | [4] |
| PL2317 | W3110 Δ*zwf*::*kan* | [4] |
| PL2342 | W3110 Δ*glgB*::*kan* | [4] |
| PL2343 | W3110 Δ*glgC*::*kan* | [4] |
| PL2340 | W3110 Δ*rfbA*::*kan* | [4] |
| PL2341 | W3110 Δ*rfbB*::*kan* | [4] |
| PL3236 | BW25113 Δ*galF*::*kan* | [4] |
| BH317 | BH141 Δ*pgm*::*frt* | BH141/pCP20 |
| BH265 | PL2455 Δ*opgH*::*frt* | PL2455/pCP20 |
| BH666 | MG1655 Δ*rcsB*::*kan* | [4] |
| BH183 | MG1655 Δ*rpoS*::*tet* | [5] |
| PL3035 | MG1655 Δ*minCDE*::*kan* | [6] |
| PL3036 | MG1655 Δ*slmA*::*kan* | [7] |
| PL2974 | MG1655 Δ*clpX*::*kan* | [4] |
| BH657 | MG1655 *opgH*::*opgH*-*6xhis* | Recombineering |
| BH663 | MG1655 *opgH* (*PIC249AIA*) | Recombineering |
| BH270 | BH265 Δ*galU*::*kan* | P1(PL2449) x BH265 |
| BH271 | BH265 Δ*opgG*::*kan* | P1(PL2450) x BH265 |
| BH485 | BH265 Δ*pgm*::*kan* | P1(BH141) x BH265 |
| BH667 | BH317 Δ*rcsB*::*kan* | P1(BH666) x BH141 |
| BH668 | BH265 Δ*rcsB*::*kan* | P1(BH666) x BH265 |
| BH192 | BH183 Δ*pgm*::*kan* | P1(BH141) x BH183 |
| BH194 | BH183 Δ*galU*::*kan* | P1(PL2449) x BH183 |
| BH206 | BH183 Δ*opgH*::*kan* | P1(PL2455) x BH183 |
| BH652 | BH317 Δ*minCDE*::*kan* | P1(PL3035) x BH317 |
| BH653 | BH265 Δ*minCDE*::*kan* | P1(PL3035) x BH265 |
| BH639 | BH317 Δ*slmA*::*kan* | P1(PL3036) x BH317 |
| BH640 | BH265 Δ*slmA*::*kan* | P1(PL3036) x BH265 |
| BH641 | BH317 Δ*clpX*::*kan* | P1(PL2974) x BH317 |
| BH642 | BH265 Δ*clpX*::*kan* | P1(PL2974) x BH265 |
| PL2452 | MG1655 *ftsZ84* (*tet*) | [8] |
| BH173 | PL2452 Δ*pgm*::*kan* | P1(BH141) x PL2452 |
| BH175 | PL2452 Δ*galU*::*kan* | P1(PL2449) x PL2452 |
| BH352 | PL2452 Δ*opgH*::*kan* | P1(PL2455) x PL2452 |
| BH330 | MG1655 *P_lac_*::*gfp-ftsZ* (*bla*) | [9] |
| BH331 | BH141 *P_lac_*::*gfp-ftsZ* (*bla*) | P1(BH330) x BH141 |
| BH647 | PL2455 *P_lac_*::*gfp-ftsZ* (*bla*) | P1(BH330) x PL2455 |
| PL644 | PL3035 *P_lac_*::*gfp-ftsZ* (*bla*) | P1(BH330) x PL3035 |
| BH654 | BH652 *P_lac_*::*gfp-ftsZ* (*bla*) | P1(BH330) x BH652 |
| BH669 | BH653 *P_lac_*::*gfp-ftsZ* (*bla*) | P1(BH330) x BH653 |
| BH645 | PL3036 *P_lac_*::*gfp-ftsZ* (*bla*) | P1(BH330) x PL3036 |
| BH648 | BH639 *P_lac_*::*gfp-ftsZ* (*bla*) | P1(BH330) x BH639 |
| BH649 | BH640 *P_lac_*::*gfp-ftsZ* (*bla*) | P1(BH330) x BH640 |
| BH647 | PL2974 *P_lac_*::*gfp-ftsZ* (*bla*) | P1(BH330) x BH647 |
| BH650 | BH641 *P_lac_*::*gfp-ftsZ* (*bla*) | P1(BH330) x BH650 |
| BH651 | BH642 *P_lac_*::*gfp-ftsZ* (*bla*) | P1(BH330) x BH651 |
| PL3180^c^ | W3110 *ftsZ*::*kan* | [10] |

**^a^** *P_lac_* indicates the lactose promoter. *bla, tet* and *kan* indicate ampicillin, tetracycline and kanamycin resistance cassettes. The *kan* resistance cassettes are flanked by *frt* sites for excision by the FLP recombinase leaving a *frt* scar sequence.

**^b^** P1 transduction is succinctly described as: P1(donor strain) x recipient strain.

^c^ Supplemented with pKG110-*ftsZ.*

**TABLE S3 REFERENCES**

1. Guyer MS, Reed RR, Steitz JA, Low KB (1981) Identification of a sex-factor-affinity site in *E. coli* as gamma delta. Cold Spring Harb Symp Quant Biol 45 Pt 1: 135-140.

2. Bachmann BJ (1972) Pedigrees of some mutant strains of *Escherichia coli* K-12. Bacteriol Rev 36: 525-557.

3. Wood WB (1966) Host specificity of DNA produced by *Escherichia coli*: bacterial mutations affecting the restriction and modification of DNA. J Mol Biol 16: 118-133.

4. Baba T, Ara T, Hasegawa M, Takai Y, Okumura Y, et al. (2006) Construction of *Escherichia coli* K-12 in-frame, single-gene knockout mutants: the Keio collection. Mol Syst Biol 2: 2006 0008.

5. Zhou Y, Gottesman S (2006) Modes of regulation of RpoS by H-NS. J Bacteriol 188: 7022-7025.

6. Bernhardt TG, de Boer PA (2005) SlmA, a nucleoid-associated, FtsZ binding protein required for blocking septal ring assembly over chromosomes in *E. coli*. Mol Cell 18: 555-564.

7. Cho H, McManus HR, Dove SL, Bernhardt TG (2011) Nucleoid occlusion factor SlmA is a DNA-activated FtsZ polymerization antagonist. Proc Natl Acad Sci U S A 108: 3773-3778.

8. Belhumeur P, Drapeau GR (1984) Regulation of cell division in *Escherichia coli*: properties of new *ftsZ* mutants. Mol Gen Genet 197: 254-260.

9. Hale CA, de Boer PA (1999) Recruitment of ZipA to the septal ring of *Escherichia coli* is dependent on FtsZ and independent of FtsA. J Bacteriol 181: 167-176.

10. Shiomi D, Margolin W (2008) Compensation for the loss of the conserved membrane targeting sequence of FtsA provides new insights into its function. Mol Microbiol 67: 558-569.
