# Supplementary material for: A Moonlighting Enzyme Links Escherichia coli Cell Size with Central Metabolism
Source: PLoS Genet. 2013 Jul 25;9(7):e1003663. doi: 10.1371/journal.pgen.1003663 (PMC3723540; doi:10.1371/journal.pgen.1003663)
Supplement: Table S4 — Bacterial plasmids used in this study. (DOC) [file pgen.1003663.s014.doc]

**Table S4.** Bacterial plasmids used in this study.

| **Plasmid** | **Relevant genotype^a^** | **Source** |
| --- | --- | --- |
| pDR155 | *P_lac_*::*minD* (*bla*) | [1] |
| pBH228 | *P_ara_*::*thio-6xhis* (*bla*) | Invitrogen |
| pBH421 | *P_lac_*::*pgm-gfp* (*cat*) | [2] |
| pBH422 | *P_lac_*::*galT-gfp* (*cat*) | [2] |
| pBH423 | *P_lac_*::*galE-gfp* (*cat*) | [2] |
| pBH424 | *P_lac_*::*opgG-gfp* (*cat*) | [2] |
| pBH425 | *P_lac_*::*opgH-gfp* (*cat*) | [2] |
| pBH426 | *P_lac_*::*ugd-gfp* (*cat*) | [2] |
| pBH427 | *P_lac_*::*galU-gfp* (*cat*) | [2] |
| pBH429 | *P_lac_*::*otsA-gfp* (*cat*) | [2] |
| pBH494 | *P_lac_*::*opgH (PIC249AIA)-gfp* (*cat*) | This study |
| pBH536 | *P_ara_*::*thio-pgm-6xhis* (*bla*) | This study |
| pBH537 | *P_ara_*::*thio-galU-6xhis* (*bla*) | This study |
| pBH538 | *P_ara_*::*thio-opgH(1-414)-6xhis* (*bla*) | This study |
| pBH539 | *P_ara_*::*thio-opgH(573-1542)-6xhis* (*bla*) | This study |
| pBH540 | *P_ara_*::*thio-opgH(2106-2455)-6xhis* (*bla*) | This study |
| pBH541 | *P_ara_*::*thio-opgH-6xhis* (*bla*) | This study |
| pBH580 | *P_ara_*::*thio-opgH(1-201)-6xhis* (*bla*) | This study |
| pBH581 | *P_ara_*::*thio-opgH(204-414)-6xhis* (*bla*) | This study |
| pBH582 | *P_ara_*::*thio-opgH(249-414)-6xhis* (*bla*) | This study |
| pBH583 | *P_ara_*::*thio-opgH(294-414)-6xhis* (*bla*) | This study |
| pBH584 | *P_ara_*::*thio-opgH(339-414)-6xhis* (*bla*) | This study |
| pBH585 | *P_ara_*::*thio-opgH(204-369)-6xhis* (*bla*) | This study |
| pBH586 | *P_ara_*::*thio-opgH(204-336)-6xhis* (*bla*) | This study |
| pBH587 | *P_ara_*::*thio-opgH(204-270)-6xhis* (*bla*) | This study |
| pBH588 | *P_ara_*::*thio-opgH(249-336)-6xhis* (*bla*) | This study |
| pBH589 | *P_ara_*::*thio-opgH(249-303)-6xhis* (*bla*) | This study |
| pBH608 | *P_ara_*::*thio-opgH(*Δ*249-303)-6xhis* (*bla*) | This study |
| pBH616 | *P_T7_*::*opgH-intein* (*bla*) | This study |
| pBH664 | *P_ara_*::*thio-opgH(573-2544)-6xhis* (*bla*) | This study |
| pBH671 | *P_ara_*::*thio-opgH(PIC249AIA)-6xhis* (*bla*) | This study |

**^a^** *P_lac_*, *P_ara_*, or *P_T7_* indicate the lactose, arabinose, or the phage T7 promoters. *bla* and *cat* indicate ampicillin and chloramphenicol resistance cassettes.

**SUPPORTING INFORMATION TABLE 4 REFERENCE**

1. Raskin DM, de Boer PA (1999) Rapid pole-to-pole oscillation of a protein required for directing division to the middle of *Escherichia coli*. Proc Natl Acad Sci U S A 96: 4971-4976.

2. Kitagawa M, Ara T, Arifuzzaman M, Ioka-Nakamichi T, Inamoto E, et al. (2005) Complete set of ORF clones of *Escherichia coli* ASKA library (a complete set of *E. coli* K-12 ORF archive): unique resources for biological research. DNA Res 12: 291-299.

3. Nakano S, Zheng G, Nakano MM, Zuber P (2002) Multiple pathways of Spx (YjbD) proteolysis in *Bacillus subtilis*. J Bacteriol 184: 3664-3670.
