# Supplementary material for: A Moonlighting Enzyme Links Escherichia coli Cell Size with Central Metabolism
Source: PLoS Genet. 2013 Jul 25;9(7):e1003663. doi: 10.1371/journal.pgen.1003663 (PMC3723540; doi:10.1371/journal.pgen.1003663)
Supplement: Text S1 — A description of media conditions, strain construction, and auxiliary methods. (DOC) [file pgen.1003663.s015.doc]

**TEXT S1**

**Media, plasmid and strain construction**

Antibiotics were used at 100µg/ml ampicillin (Amp), 25µg/ml chloramphenicol (Cm), 12.5µg/ml tetracycline (Tet), and 25 or 50µg/ml kanamycin (Kan). Strains and plasmids employed are listed in Tables S3 and S4. Plasmid DNA and PCR fragments were puriﬁed using the Wizard miniprep or gel and PCR clean-up kits (Promega). PCR reactions used MG1655 genomic DNA as template and the high-fidelity VENT polymerase (NEB). A brief description of plasmid and strain construction is given below. Oligonucleotides used in PCR reactions are highlighted in bold, italics, or underlined to indicate initial annealing regions, restriction enzyme sites, and/or sites of mutagenesis, respectively. Recombineering strategies used plasmids (pKD3, pKD4, pKD46, & pCP20) from [1]. Many of the constructs were cloned into a pBAD/TOPO Thio-Fusion expression vector (Invitrogen) with a 5’ NcoI site.

**pBH494**

*P_lac_*::*opgH(PIC249AIA)-gfp* was constructed by site directed mutagenesis of pBH425 (*P_lac_*::*opgH-gfp*) using the Quikchange method (Stratagene), the oligo

**CGCACGGCGTTGATCATG**GCT**ATC**GCT**AACGAAGACGTGAAC**, and the complementing oligo.

**pBH536**

*pgm* was amplified using oligos *CCATGG***GCAATCCACAATCGTGCA**

and **TTACGCGTTTTTCAGAACTTCGC**. The resulting 1638bp amplicon was TOPO cloned into the ThioFusion expression vector.

**pBH537**

*galU* was amplified using oligos *CCATGG***ATGGCTGCCATTAATACGAAAGTC** and **TTACTTCTTAATGCCCATCTCTTC**. The resulting 906bp amplicon was TOPO cloned into the ThioFusion expression vector.

**pBH538**

The N-terminus of *opgH* was amplified using oligos *CCATGG***ATGAATAAGACAACTGAGTACATT** and **ACGGCGGATGGTACCGACG**. The resulting 414bp amplicon was TOPO cloned into the ThioFusion expression vector.

**pBH539**

The sugar-binding domain (middle cytoplasmic domain) of *opgH* was amplified using oligos *CCATGG***ACGGCGTTAATGGGCTTCCTGCAA** and **CATACCCTTCACCAGGAACAGACG**. The resulting 909bp amplicon was TOPO cloned into the ThioFusion expression vector.

**pBH540**

The C-terminus of *opgH* was amplified using oligos *CCATGG***CGTGCCACCGTTGGTCTGCGCACC** and **CGCATCCGGTTTACGCAATGC**. The resulting 438bp amplicon was TOPO cloned into the ThioFusion expression vector.

**pBH541**

*opgH* was amplified using oligos 5’*CCATGG***ATGAATAAGACAACTGAGTACATT** and 5’**CGCATCCGGTTTACGCAATGC**. The resulting 2455bp amplicon was TOPO cloned into the ThioFusion expression vector.

**pBH580**

The first half of the N-terminus (residues 1-67) of *opgH* was amplified using oligos *CCATGG***ATGAATAAGACAACTGAGTACATT** and **TCCATCAGCAAGTGAATCTGGCCA**. The resulting 201bp amplicon was TOPO cloned into the ThioFusion expression vector.

**pBH581**

The second half of the N-terminus (residues 68-138) of *opgH* was amplified using oligos *CCATGG***CAGTTAATTAAAGACGACGAAGGG** and **ACGGCGGATGGTACCGACG**. The resulting 201bp amplicon was TOPO cloned into the ThioFusion expression vector.

**pBH582**

*opgH* was amplified using oligos *CCATGG***GAAGCAAAACGCTCCTCGATG** and **ACGGCGGATGGTACCGACG**. The resulting 165bp amplicon (coding for residues 83-138) was TOPO cloned into the ThioFusion expression vector.

**pBH583**

*opgH* was amplified using oligos *CCATGG***GGCCGTTTCTGGGATCGC** and **ACGGCGGATGGTACCGACG**. The resulting 120bp amplicon (coding for residues 98-138) was TOPO cloned into the ThioFusion expression vector.

**pBH584**

*opgH* was amplified using oligos *CCATGG***CCGCGCTATCTGGCTCGTTTG** and **ACGGCGGATGGTACCGACG**. The resulting 75bp amplicon (coding for residues 113-138) was TOPO cloned into the ThioFusion expression vector.

**pBH585**

*opgH* was amplified using oligos *CCATGG***CAGTTAATTAAAGACGACGAAGGG** and **CTCTTCTTTGGTCAAACGAGCCAG**. The resulting 165bp amplicon (coding for residues 68-123) was TOPO cloned into the ThioFusion expression vector.

**pBH586**

*opgH* was amplified using oligos *CCATGG***CAGTTAATTAAAGACGACGAAGGG** and CGTGCATCGCGTCCACG . The resulting 165bp amplicon (coding for residues 68-112) was TOPO cloned into the ThioFusion expression vector.

**pBH587**

*opgH* was amplified using oligos *CCATGG***CAGTTAATTAAAGACGACGAAGGG** and AAACATCGAGGAGCGTTTTGC. The resulting 165bp amplicon (coding for residues 68-90) was TOPO cloned into the ThioFusion expression vector.

**pBH588**

*opgH* was amplified using oligos *CCATGG***GAAGCAAAACGCTCCTCGATG** and **CGTGCATCGCGTCCACG**. The resulting 87bp amplicon (coding for residues 83-112) was TOPO cloned into the ThioFusion expression vector.

**pBH589**

*opgH* was amplified using oligos *CCATGG***CAGTTAATTAAAGACGACGAAGGG** and **CGTGACATCGCGTCCACG**. The resulting 165bp amplicon (coding for residues 83-101) was TOPO cloned into the ThioFusion expression vector.

**pBH608**

*opgH* was amplified using oligos *CCATGG***ATGAATAAGACAACTGAGTACATT** and ATA*GGATCC* **TTCTGGCATCGCCTTCAGCTG** as well as ATA*GGATCC***GTAGGCCGTTTCTGGGATC** and **ACGGCGGATGGTACCGACG**

to create amplicons bp1-249 and bp303-414, respectively. The amplicons were then digested with BamHI, ligated, amplified using oligos ***CCATGG*ATGAATAAGACAACTGAGTACATT** and **ACGGCGGATGGTACCGACG.** The resulting 375bp amplicon was TOPO cloned into the ThioFusion expression vector.

**pBH664**

A *opgH* construct excluding the N-terminus and first two transmembrane domain was constructed by using oligos *CCATGG***ACGGCGTTAATGGGCTTCCTGCAA**

and **CGCATCCGGTTTACGCAATGC**. The resulting 1971bp amplicon was TOPO cloned into the ThioFusion expression vector (Invitrogen).

**pBH671**

*opgH* was amplified from pBH494 using oligos *CCATGG***ATGAATAAGACAACTGAGTACATT** and **CGCATCCGGTTTACGCAATGC**. The resulting 2455bp amplicon was TOPO cloned into the ThioFusion expression vector.

**pBH616**

The N-terminus of *opgH* was amplified using oligos ATT*CCATGG*TT**ATGAATAAGACAACTGAGTACATT** and ATT*CCCGGG***ACGGCGGATGGTACCGACG**. The resulting fragment was digested with NcoI and SmaI and ligated into pTYB4 (Invitrogen) yielding an in-frame *opgH^N^*-intein fusion.

**BH643**

An insertion into *opgG* was achieved using lambda red recombination [1]. Amplifying using oligo CGTTGGTTGAGTGCTGCAGTAATGTTAACCCTGTATACA**GTGTAGGCTGGAGCTGCTTC** with

GCTGGTTTGCGCTGTGACCGGGTATCCTCTGGCAGTTT**ATGGGAATTAGCCATGGTCC** using pKD3 as template created a PCR product containing a chloramphenicol resistance cassette flanked with homologous regions to *opgG*. The resulting amplicon was transformed into BH249 (MG1655 + pKD46) to knockout *opgG*. The *cm^R^* cassette was removed using pCP20.

**BH663**

*opgH* was amplified using pBH494 (*P_lac_*::*opgH*(PIC249AIA)-*gfp*) as template with oligos **CATCCGGTTTACGCAATGC** and AAGCAGCTCCAGCCTACA**TTATTGCGAAGCCGCATCCG**. Separately, a kanamycin resistance cassette was amplified with a homologous region 36 bp distal to the 3’ end of *opgH* from pKD4 [1] using oligos **TGTAGGCTGGAGCTGCTT** and

TGCAAAATCAATAAATTGCAGGAACGATGTA**TGACATGGGAATTAGCCATGGTC**. The two PCR products were then SOE’d together by PCR using **CATCCGGTTTACGCAATGC** and TGCAAAATCAATAAATTGCAGGAACGATGTA**TGACATGGGAATTAGCCATGGTC,** which created a PCR product of ~4.0kb. This was then electroporated into WT MG1655 cells harboring the lambda red plasmid pKD46 to facilitate chromosomal integration. Kan^R^ colonies were then screened for the mutation by sequencing.

**Chemotaxis assay**

For our purposes chemotaxis was used as an indirect assessment of OPG production. OPGs are monitored by the Rcs phosphorelay system. An absence of OPGs in the periplasm causes a down-regulation of flagellar synthesis and chemotaxis [2]. Early-log cultures (OD_600_ 0.15-0.4) were normalized to optical density. Subsequently, 1µl of culture was stabbed into a swarm plate (1% tryptone, 0.5% NaCl, and 0.3% agar) ± inducer. Plates were incubated at 30ºC for 20 hours and imaged. The diameter was calculated by the average of three measurements.

**Quantitative Real-Time PCR**

*E. coli* cells were harvested for RNA in early-log (an OD_600_ of ~0.25) with the [RiboPure™](http://www.ambion.com/catalog/CatNum.php?1924) Kit (Ambion), treated with the Turbo DNA-Free Kit™ (Ambion), and reverse transcribed for 1 hour at 42˚C using the RETROscript® Kit (Ambion). Template was diluted 10-fold and added to iTaq SYBR Green Supermix (Bio-Rad) and amplified with primers to either *opgH* (**GGTGCTGTTGTTCCTGCCGAAGTTATTG** and **CAGCAACGATAATGTAACGCGCCAGAAG**) or *ftsZ* (**ATTTGGGTATCCTGACCGTTGCTG** and **ACTTTCAGCAGTTTGTCGTTCGGG**) using an Applied Biosystems model 7500 thermocycler. Results were analyzed using the comparative Pfaffl method [3].

**Chromosomal origins per cell**

Determination of *oriC* per cell was determined by evaluating the flow cytometry profiles after replication run out identically to described in [4].

**Supporting TEXT References**

1. Datsenko KA, Wanner BL (2000) One-step inactivation of chromosomal genes in *Escherichia coli* K-12 using PCR products. Proc Natl Acad Sci U S A 97: 6640-6645.

2. Girgis HS, Liu Y, Ryu WS, Tavazoie S (2007) A comprehensive genetic characterization of bacterial motility. PLoS Genet 3: 1644-1660.

3. Pfaffl MW (2001) A new mathematical model for relative quantification in real-time RT-PCR. Nucleic Acids Res 29: e45.

4. Hill NS, Kadoya R, Chattoraj DK, Levin PA (2012) Cell size and the initiation of DNA replication in bacteria. PLoS Genet 8: e1002549.
